# Internal Laser Writing of High-Aspect-Ratio Microfluidic Structures in Silicate Glasses for Lab-on-a-Chip Applications

**DOI:** 10.3390/mi8020059

**Published:** 2017-02-16

**Authors:** Ya Cheng

**Affiliations:** 1 State Key Laboratory of Precision Spectroscopy, East China Normal University, Shanghai 200062, China; ycheng@phy.ecnu.edu.cn; Tel.: +86-21-6991-8546; Fax: +86-21-6991-8021; 2 State Key Laboratory of High Field Laser Physics, Shanghai Institute of Optics and Fine Mechanics, Chinese Academy of Sciences, P.O. Box 800-211, Shanghai 201800, China

**Keywords:** internal processing, femtosecond laser, silicate glass, microfluidics, nanofluidics, lab-on-a-chip

## Abstract

Femtosecond laser direct writing is unique in allowing for fabrication of 3D micro- and nanofluidic structures, thereby enabling rapid and efficient manipulation of fluidic dynamics in 3D space to realize innovative functionalities. Here, I discuss the challenges in producing fully functional and highly integrated 3D micro- and nanofluidic systems with potential applications ranging from chemical and biological analyses to investigations of nanofluidic behaviors. In particular, I review the achievements we have made in the past decade, which have led to 3D microchannels with controllable cross-sectional profiles and large aspect ratios, 3D nanofluidic channels with widths of several tens of nanometers, and smooth inner walls with roughness on the order of ~1 nm. Integration of the microfluidics with other functional microcomponents including microoptics and microelectrodes will also be discussed, followed by conclusions and the future perspective.

## 1. Introduction

Manipulation of liquids in micro- and nanoscale environments with high precision and flexibility is highly desirable in many fields such as chemistry, biology, medicine, photonics, and fluidic physics [1]. Today, microfluidics has become an important interdisciplinary subject of research and is undergoing rapid development [2]. This is largely due to the recent technical advances in fabricating microfluidic devices, making it possible to produce micro- and nanoscale channels on the surface of various materials. The most popular technique for fabricating microfluidics is soft lithography, which was developed by Whitesides et al. [3]. Soft lithography typically requires masters fabricated on silicon wafers by conventional photolithography to serve as the molds. By pouring liquid polydimethylsiloxane (PDMS) prepolymer onto the master and curing the prepolymer at ~70 °C, a PDMS replica can be formed after the cured prepolymer is peeled off from the master. To form enclosed microfluidic channels, the replica must be sealed by a flat surface. Soft lithography is an inherently 2D technology due to the photolithography step. Consequently, it has limited ability and flexibility in terms of producing 3D microstructures with arbitrary geometries. Realizing 3D microfluidic networks for achieving enhanced functionalities remains largely unexplored.

At present, the dominating technology that allows for straightforward fabrication of 3D micro- and nanofluidics buried in glass without any stacking and bonding procedures is femtosecond laser direct writing (FLDW). The strategy to overcome the difficulty of achieving the internal processing capability is to tightly focus the femtosecond laser beam into substrate materials which are transparent at the laser wavelengths. The ultrashort pulse durations of the laser pulses can generate extremely high peak intensities inside glass even with moderate pulse energies, leading to highly nonlinear multiphoton absorption efficiently confined to a region near the focal point [4]. However, for fabrication of micro- and nanofluidic structures, the materials not only need to be modified with their physical or chemical properties, they also need to be selectively removed to form the hollow channels and chambers within the substrates. This creates the major challenge in the fabrication and application of 3D micro- and nanofluidic structures.

Currently, two methods have been well established for overcoming the difficulty in the selective removal of materials from the laser affected regions. The first method relies on selectively promoting the chemical etching rate in the laser modified areas, which is called femtosecond laser assisted chemical wet etching [5,6]. Hollow microfluidic structures are produced by preferentially removing the laser-modified materials with the chemical wet etching. Unlike this method, which involves essential chemical procedures and therefore unavoidably requires postprocessing after the laser exposure, the second method produces hollow channels by performing femtosecond laser 3D drilling from the rear surface of the glass in contact with distilled water. In this manner, the water introduced into the microchannel can help to remove the ablated debris [7,8]. Remarkably, both the techniques have shown great flexibility in producing microfluidic structures with arbitrary 3D geometries, based on which a broad variety of innovative microfluidic systems have been produced. The technical details of the two approaches as well as their applications in manufacturing integrated microfluidic devices have been well-documented in a few review articles [9,10,11]. 

In the following, I will first discuss the major challenges in promoting 3D microfluidics in real-world applications and several solutions we have developed for overcoming the challenges (Section 2). I will then present a few examples of functional microfluidic systems manufactured by FLDW (Section 3). Finally, conclusion and future perspective will be given (Section 4).

## 2. Challenges in Real World Applications and Current Solutions

The unique advantages of fabricating micro- and nanofluidic structures with FLDW are the 3D fabrication capability, flexibility in controlling the cross-sectional shape of the channels, and promising potential of multifunctional integration. All of these advantages have been conceptually proven in the past two decades. Surprisingly, 3D microfluidic structures have not seen widespread use in practice. To understand the situation, it should be noted that for many micro- and nanofluidic applications, three typical requirements exist, which are listed as follows.

i The microchannels need to be sufficiently long to ensure completion of the chemical reactions during the transportation of the liquid samples.
ii The inner walls of microchannels need to be sufficiently smooth in order not to disturb the fluidic dynamics or damage the biological samples (e.g., the membranes of living cells).
iii The fabrication resolution should be as high as possible to produce channels whose feature sizes can range from micrometer to nanometer scales.

It should be noted that the first two requirements can be readily fulfilled with optical-lithography-based fabrication technologies, in which the open channels are first fabricated on the top surfaces of substrates and then sealed with a cover plate. For the third requirement, although the resolution of optical lithography is limited to micrometer scale due to the optical diffraction limit, nanofluidic channels can still be fabricated on the surfaces of substrates by electron beam writing. We shall stress again that the lithographic approaches are intrinsically limited to 2D planar geometries, and thus cannot replace FLDW when 3D geometries are the only choice. 

Unfortunately, the above three requirements are difficult to fulfill with the FLDW. Although a perfect solution to overcome all these challenges at once has not been found, we have developed a few techniques to resolve the issues individually.

### 2.1. Fabrication of Homogeneous 3D Microfluidic Channels of Great Lengths

To produce buried microfluidic channels in glass with diameters of tens of micrometers and lengths at a 10-cm scale, it is necessary to find a way to efficiently remove the materials in the thin channels of very large aspect ratios (i.e., the ratios between the lengths and the transverse dimensions of the channels). This is difficult because when the microchannel grows longer and longer during its formation, it will be harder and harder to evacuate the debris from the bottom of the thin channels. This happens when fabricating the microfluidic channels with both femtosecond laser assisted chemical etching and water assisted femtosecond laser 3D drilling. The idea of using a porous glass as the substrate for producing 3D microfluidics was born in our group in 2006, because it was expected that ablating inside the porous glass could lead to microexplosion within the bulk, which should produce hollow voids due to the densification in the surrounding area. Hollow microfluidic channels could therefore be formed by connecting the hollow voids along a 3D trajectory defined by the motion of the focal spot. In this way, as it would be unnecessary to remove the debris from the thin channels, channels of infinite lengths should be achievable. Interestingly, it was found later that porous glass does allow for fabrication of microchannels of great lengths; however, the mechanism is different from that mentioned above and will be described below. 

At the beginning, it was very difficult to find a suitable porous material for such application. The first porous glass we tested in our experiment was produced using sol-gel technique. The glass sample was fragile from a mechanical point of view. Ablating in such porous glass easily created large cracks, and sometimes the glass even broke up during the laser irradiation. In 2010, it was found that a porous glass produced by Chen et al. could serve as an ideal substrate material for fabricating microfluidic channels of almost arbitrary lengths and 3D geometries [12]. The porous glass was produced by removing the borate phase from phase-separated alkali-borosilicate glass in hot acid solution [13]. The composition of the porous glass is approximately 95.5SiO_2_-4B_2_O_3_-0.5Na_2_O (wt %). The pores with a mean size of ~10 nm are distributed uniformly in the glass and occupy 40% volume of the glass. 

**Figure 1:**
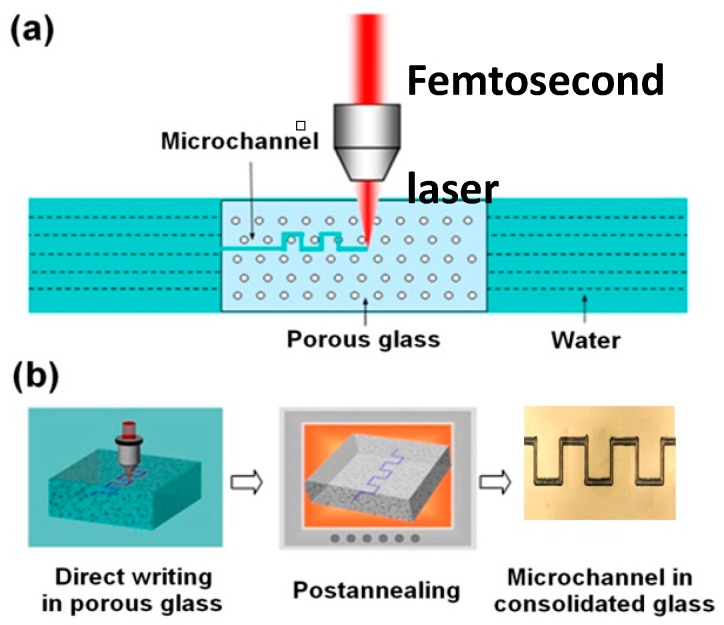
(**a**) Schematic of experimental setup; and (**b**) flow diagram for the fabrication process.

The schematic of the water-assisted FLDW inside porous glass is illustrated in Figure 1a. The porous glass was immersed in distilled water; otherwise, the glass would be opaque due to the strong scattering from the pores. Since the pores in the porous glass form a 3D connective network which allows liquid to flow through, water bubbles can be produced by laser ablation near the focus, which helps remove the debris when the bubbles are driven out from the channel by the shockwave [14]. The laser beam was focused into the sample, and 3D microchannels could be fabricated by scanning the sample according to a preprogrammed pattern. However, the microchannels produced immediately after the laser ablation were useless for microfluidic application, as any liquids injected into the channels would leak into the surrounding pores. Therefore, the fabricated sample was subjected to an annealing at a temperature of 1150 °C. During the annealing, the nanopores collapsed and the porous glass gradually consolidated into a compact glass, as illustrated in Figure 1b. After the annealing, the microfluidic channels could contain liquids without any leakage.

**Figure 2:**
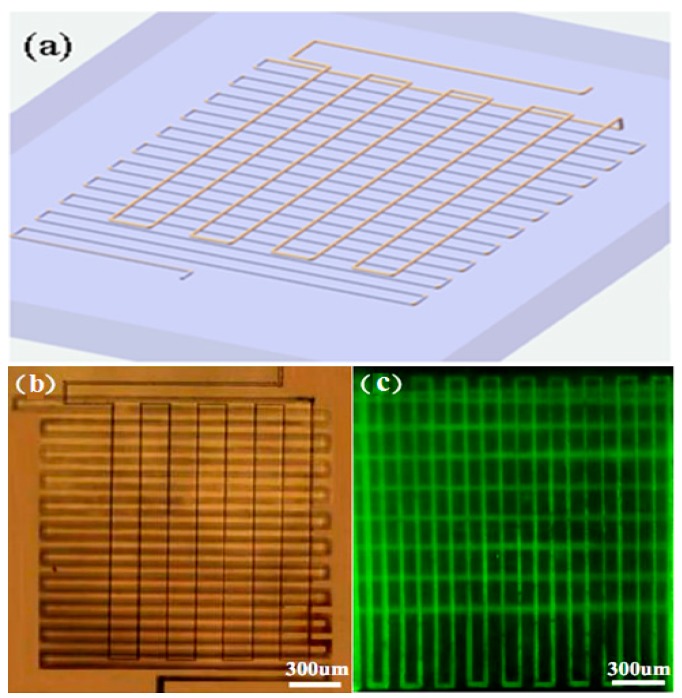
(**a**) Schematic diagrams of the 3D microchannel; and (**b**) optical micrograph of a 3D microchannel arranged in a compact space before the postannealing of the fabricated sample; (**c**) the fluorescence microscope image of the 3D microchannel filled with a solution of fluorescein. The volume of the substrate is reduced by ~39% as a result of the collapse of the nanopores.

The technique allows us to fabricate microfluidic channels of great lengths, whereas the microchannels remain homogeneous without any visible taper (i.e., the microfluidic channels fabricated with chemical etching typically have a taper due to the longer etching durations near the openings) [15]. Figure 2a shows the schematic diagram of a 3D microchannel. The multi-layer microchannel in 3D space was fabricated by translating the sample in a line-by-line and layer-by-layer manner, and the scan started from the lower layer in order to avoid the extra aberration induced by the structures fabricated beforehand in the upper layers. As indicated by its top view micrograph, the 3D microchannel in Figure 2b has a total length of ~6.6 cm. Remarkably, such a long channel was compactly folded within a tiny volume of only ~120 nL. The long channel was measured to have a width of ~10 μm and a height of ~12 μm, resulting in a high aspect ratio of ~6000. The vertical distance between the two layers was ~30 μm according to our design. It took ~3 h to fabricate the 3D microfluidic channel as shown in Figure 2b. In fact, longer microchannels can be fabricated using this technique without much difficulty whereas a longer fabrication time will be required. After the 3D microchannel was filled with a solution of fluorescein and excited with a mercury lamp, a fluorescence microscope image was taken as shown in Figure 2c. The image clearly indicates that the channel is a through structure which allows fluids to flow in and out.

### 2.2. Fabrication of Microfluidic Channels of Smooth Inner Wall

Typically, the microfluidic channels fabricated by femtosecond laser-assisted chemical etching or water-assisted femtosecond laser drilling have an inner surface roughness on the order of tens of nanometers, which is undesirable for many applications such as investigation of micro- and nanofluidic physics and analysis of living cells. For thick channels of relatively large diameters (e.g., a few hundred micrometers), it has been found that the surface roughness can be greatly reduced by postannealing, as the annealing will result in a controlled reflow on the inner surfaces [16]. However, for channels of smaller diameters (e.g., diameters of tens of micrometers or less), this technique is not effective (the physics behind this has not been well understood).

**Figure 3:**
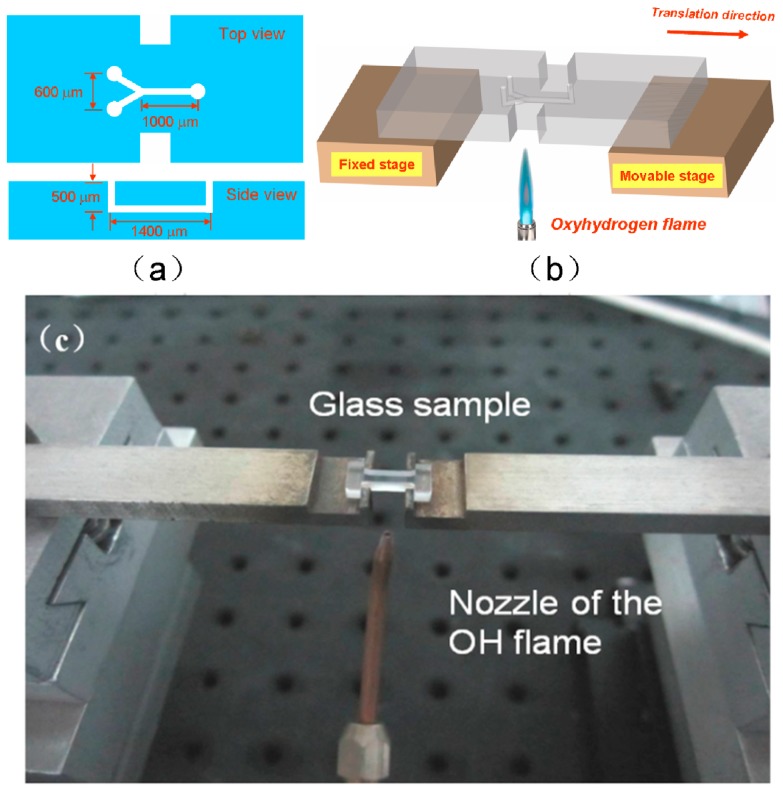
Schematics of (**a**) 3-D microfluidic channel and (**b**) the glass drawing setup; (**c**) picture of the glass drawing setup.

**Figure 4:**
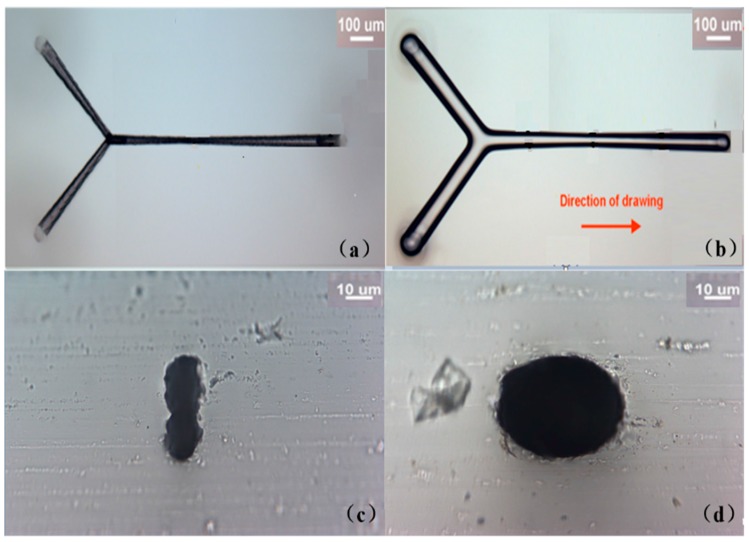
Optical micrographs of the Y-branched channels. Top view of the channels (**a**) before and (**b**) after drawing; and (**c**,**d**) cross section views of the middle regions of the channels in (a,b), respectively.

In 2010, He et al. demonstrated that homogeneous microfluidic channels with smooth inner surfaces can be fabricated by applying a glass drawing process to the channels fabricated by femtosecond laser assisted chemical etching [17]. Figure 3a shows a schematic view of a Y-shaped microchannel embedded 500 μm beneath the surface of the fused silica. The total length and width of the Y-shaped channel structure are 1400 μm and 600 μm, respectively (see Figure 4a). To facilitate drawing of glass, the two ends of fabricated sample were separately fixed on two motion stages, as illustrated in Figure 3b. The sample was heated with an oxyhydrogen flame of a diameter of ~0.5 mm, as shown in Figure 3c. Since the glass sample was softened near its melting point at ~1700 °C, we could draw the glass sample along the direction of the microfluidic channel by translating one of the two stages. The other stage remained stationary during the glass drawing. It was found that the optimum drawing speed is 1 μm/s for producing a homogeneous channel. In the experiment, the movable stage was only translated 200 μm. Despite the fact that the drawing distance was much lower than the length of channel, the change in the geometry of channel turned out to be dramatic, as shown below.

The difference in the microfluidic channels before and after the drawing process is evidenced by their respective micrographs as shown in Figure 4a,b. Obviously, the taper of fabricated microfluidic channel was severe without the drawing process. However, the tapering in the channel was significantly reduced with the glass drawing, and for the 1000 μm-long microfluidic channel demonstrated in our experiment, the taper angle was reduced from ~4.2° to ~0.8°. In addition, the cross sectional shape of the channel dramatically changed from highly elliptical to nearly circular with the help of the drawing process as shown in Figure 4c,d.

**Figure 5:**
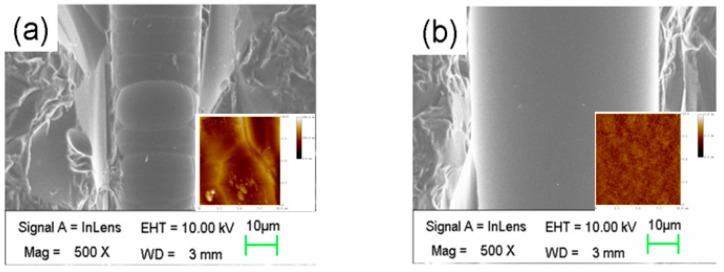
Scanning electron microscopic (SEM) images of the inner surfaces (**a**) before and (**b**) after drawing; Inset in (a,b): scanning probe micrographs of the etched inner surfaces before and after the drawing process, respectively.

Furthermore, to examine the inner surface after the drawing, we polished the samples until the microchannels were exposed. The scanning electron microscopic (SEM) images of the inner surfaces of microfluidic channels before and after the glass drawing are shown in Figure 5a,b, respectively. It is clear that after the glass drawing process, the inner surface of the microfluidic channel became very smooth. To quantitatively determine the improvement of surface roughness, scanning–probe micrographs (SPM) of the inner surfaces fabricated without and with the drawing process are shown in the insets in Figure 5a,b, respectively. In this case, flat inner surfaces were required for SPM scanning; therefore, we fabricated two microchannels with a rectangular cross section under the same experimental conditions. Amazingly, the root mean square roughness of the inner surface of the microchannel was greatly reduced from 50.3 nm to 0.29 nm with the drawing process.

### 2.3. Fabrication of Nanofluidic Channels

**Figure 6:**
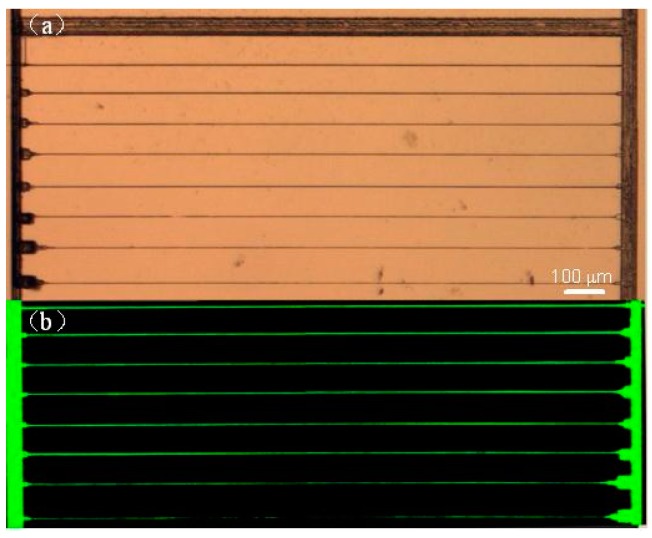
(**a**) Top view optical micrograph of submicrometer channels embedded in porous glass after the postannealing and (**b**) fluorescence microscope image of the postannealed submicrometer channels filled with a fluorescent dye.

To fabricate nanofluidic channels, a straightforward strategy is to employ the threshold effect in the interaction of femtosecond laser pulses with transparent materials [18,19,20]. Figure 6a shows the top view optical micrograph of submicrometer channels embedded in porous glass after the postannealing. In this experiment carried out by us, the channels was machined at ~120 nJ/pulse to produce a channel diameter of ~400 nm and length of ~2 mm. Fluorescence microscopy image of the submicrometer channels filled with a solution of fluorescein is shown in Figure 6b. The confined fluorescent solution gives clear evidence that the nanopores had all collapsed to form the consolidated substrate.

**Figure 7:**
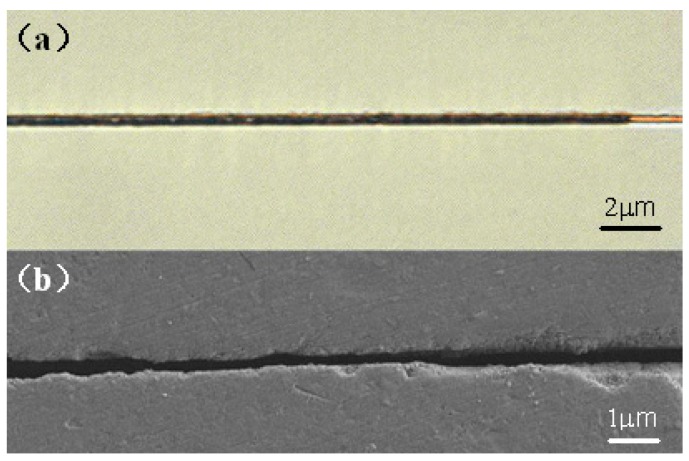
(**a**) Close-up view of submicrometer channels partially filled with water before post-annealing and (**b**) top view SEM image of the inner wall of the sub-micrometer channel written in the porous glass after post-annealing.

Figure 7a shows the closed-up view of submicrometer channels partially filled with water before the postannealing. To examine the morphology of the inner wall of sub-micrometer channel, we polished the sample from the top surface until the channel was exposed. A scanning electron microscope (SEM) image of the surface morphology of the sample after the post-annealing is shown in Figure 7b. It should be stressed that the limited length of the sub-micrometer channel is caused by the polishing plane being slightly tilted with respect to the horizontal plane where the channel lies. The image shows a through channel structure with a width of less than half micrometer throughout its length.

To further reduce the transverse dimension of the nanofluidic channels to a sub-100 nm scale, it is insufficient to only employ the threshold effect. In 2013, Liao et al. showed that nanogratings consisting of an array of hollow nano-sheets oriented along the direction of laser propagation could be formed in the porous glass with exposure to a linearly polarized femtosecond laser beam. By reducing the peak intensity of the laser pulses close to a threshold value, only a single hollow nano-sheet in the central area of the focal spot could survive, which serves as the basic element for constructing 3D nanofluidics inside glass.

**Figure 8:**
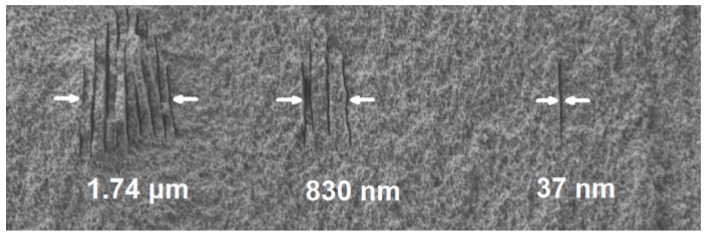
Evolution from nanograting to single nano-sheet with decreasing laser intensity.

It is well known that when the glass sample is irradiated with linearly polarized light, the nanograting structure oriented perpendicular to the laser polarization can be inscribed in various kinds of transparent materials [21]. What had not been realized at that time was that one could reliably form single, isolated nano-sheets by combining the nanograting formation and the threshold effect [20]. Figure 8 shows the cross sections of the nanogratings written at three different pulse energies at a same writing speed of 10 μm/s. At a relatively high pulse energy of 90 nJ, the number of elongated nano-sheets in the nanograting is large. After lowering the pulse energy to 70 nJ, the fabricated nanograting consists of only four nano-sheets. Meanwhile, the periodicity of the nanograting significantly increases at the decreased pulse energy as shown in Figure 8, which also helps reduce the number of nano-sheets. Finally, after reducing the pulse energy to 60 nJ (i.e., corresponding to a threshold intensity of ~1.5 × 10^14^ W/cm^2^), only the central nano-sheet survives, showing an extremely narrow width of ~37 nm. The evolution of the nanostructures in Figure 8 unambiguously manifests the central role played by the threshold effect in this phenomenon. On the other hand, in this experiment, we find little difference in the widths of individual nano-sheets written at different intensities. This means that the width of the nano-sheets is mostly determined by the physical property of the substrate but not the laser intensity, which is critical for reproducible nanofabrication [22].

**Figure 9:**
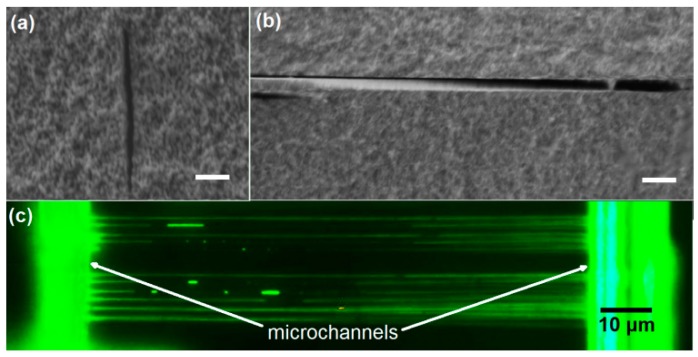
(**a**) Cross-section view and (**b**) top view SEM images of a single nanofluidic channel written in the porous glass. Scale bar = 300 nm; (**c**) fluorescence microscope image of the postannealed nanochannels filled with a fluorescent dye.

The single nano-sheets can be connected into a continuous structure along the trajectory of the scanning laser beam, enabling construction of 3D nanofluidic channels in glass. For this application, the focal spot of the femtosecond laser was slowly translated in the glass sample at a speed of 5~10 μm/s. As shown by the cross section of the nanofluidic channel in Figure 9a, the width of the nanochannel is only ~40 nm. However, the nanochannel has a height of ~1.5 μm, indicating that the longitudinal resolution is still governed by the diffraction limit of the focused laser beam. The glass sample was carefully polished until the top of the nanochannel was opened to air, as shown by its SEM image in Figure 9b. A single continuous nanochannel was clearly observed. Moreover, as further shown in Figure 9c, the entire nanofluidic channel could be filled with a dye solution, which indicates that the nanochannels fabricated with this technique are through hollow structures, i.e., all the debris produced by laser ablation had been efficiently cleaned up during the writing of the nanochannels.

## 3. Functional 3D Micro- and Nanofluidic Systems

Along with the progress made on improving the fabrication technique, efforts have also been made to realize integrated functional devices. FLDW is a versatile tool that can be used for fabricating not only microfluidic components but also optical and photonic structures as well as microelectrodes. Thus, FLDW is capable of integrating multiple functional microstructures onto a single substrate with high spatial precision.

### 3.1. Integrated Microfluidic Surface Enhanced Raman Scattering (SERS) Sensor

**Figure 10:**
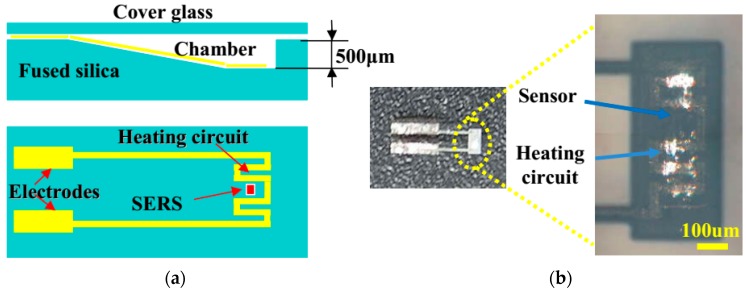
(**a**) Schematic illustration of side view (upper panel) and top view (lower panel) and (**b**) digital camera captured image of the integrated Raman bio-chip.

The first example we would like to show is a microfluidic surface enhanced Raman scattering (SERS) bio-chip, whose schematic is illustrated in Figure 10a [23]. The bio-chip, which is shown by its picture captured with a digital camera as displayed in Figure 10b, was fabricated on a fused silica glass by three steps. First, a microfluidic chamber was created by femtosecond laser ablation using amplified femtosecond laser pulses. Second, a micro-heater and two electrodes were built in the microfluidic chamber using a femtosecond laser oscillator. Lastly, the SERS substrate was fabricated in the middle area between two heating circuits in the cavity, as shown in the lower panel of Figure 10a. More details on the fabrication can be found in references [23,24]. After the absorption of R6G molecules on the substrate for 3 min, the microchamber was flushed and then filled with distilled water. Then the substrate was excited by a 532-nm laser for a few minutes in order to photo bleach the fluorescence of the dissociated R6G molecules. Raman spectra of R6G were measured with a 532-nm continuous excitation laser operated at a power of 10 mW. When the measured Raman signal became stable, the micro-electrodes were connected to a constant-current power supply by the aid of electric conductive resin and an electric current was applied on the micro-heater to heat the water in the microchamber.

**Figure 11:**
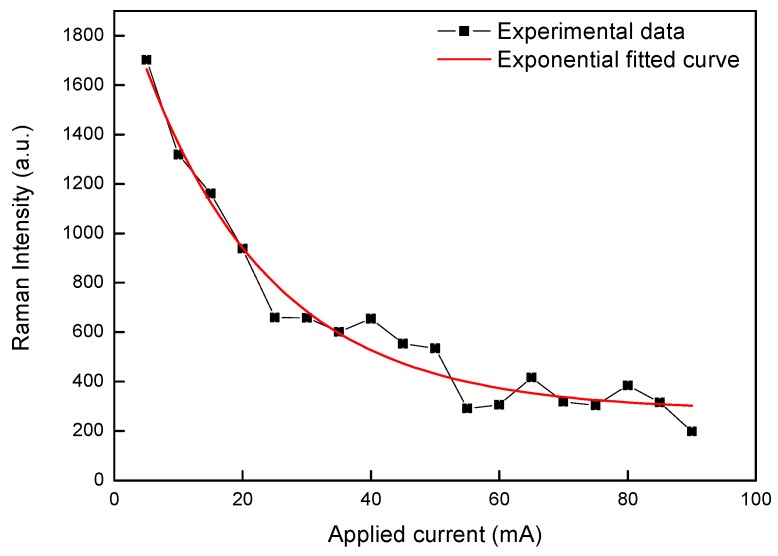
Measured Raman intensity as a function of electric current applied on the micro-heater.

The measured Raman intensity decreased with increasing electric current, as shown in Figure 11. The decrease of Raman signal with the increasing temperature in the heated microfluidic chamber could be explained by dissociation of the R6G molecules absorbed on the nanoparticles with increasing temperature. It has been reported that molecules can be “pushed out” from the SERS-active sites at the interstices or the coupling part of silver nanoparticles with increasing temperature, thus the collected Raman intensity will decrease accordingly [25]. An exponential decay of Raman signal with increasing current can be observed in Figure 11.

### 3.2. Integrated Microfluidic Whispering Gallery Mode (WGM) Microresonator (MR) Sensor

**Figure 12:**
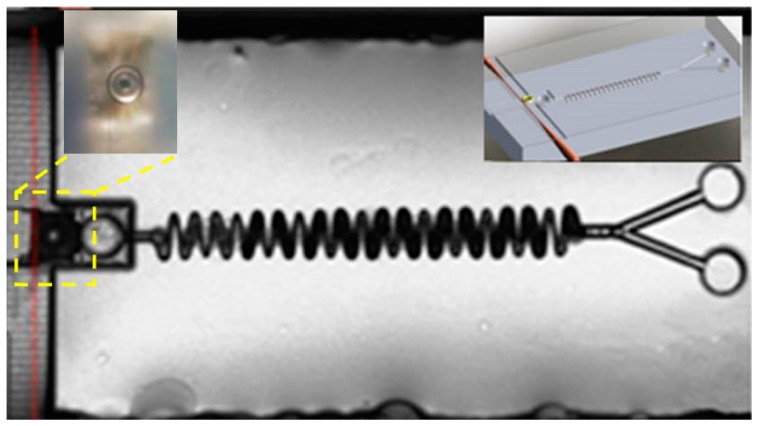
The fabricated optofluidic refractive index sensor. The fiber is indicated with the dashed red line, and the microtoroid resonator is indicted with a yellow square. Inset (**right**): Illustration of the layout of the integrated optofluidic sensor. Inset (**left**): The close-up view of the assembled microtoroid-fiber system fabricated in the microfluidic channel using a femtosecond laser.

In 2014, Song et al. demonstrated a fully integrated optofluidic sensor in which a microfluidic system and the microtoroid resonator were fabricated in a fused silica substrate together using FLDW [26,27]. The inset on the right hand side in Figure 12 schematically illustrates the design of the integrated sensor. The microfluidic system, which is composed of two branched-channels with a length of 600 μm connected with two inlets, and a sin-wave-shaped long channel with a total length of 3 mm connected with an outlet for mixing of liquid, was fabricated 300 μm beneath the glass surface by FLDW. A reservoir was fabricated near the outlet of the microfluidic channel. The mixed liquid could be injected into the reservoir from the microfluidic channel. To perform high-sensitivity on-chip refractive index sensing, a microtoroid resonator was fabricated in the middle of reservoir. The fabricated sensor in Figure 12 shows that all the components of different functionalities have been monolithically integrated in a single glass chip. The close-up view image of the integrated toroid-fiber system is shown in the inset on the left hand size in Figure 12. The fiber taper is too thin and can hardly be seen due to the limited resolutions chosen for obtaining a sufficiently large field-of-view to accommodate the whole sensor. For this reason, the fiber taper is indicated with the red dashed line in Figure 12. 

**Figure 13:**
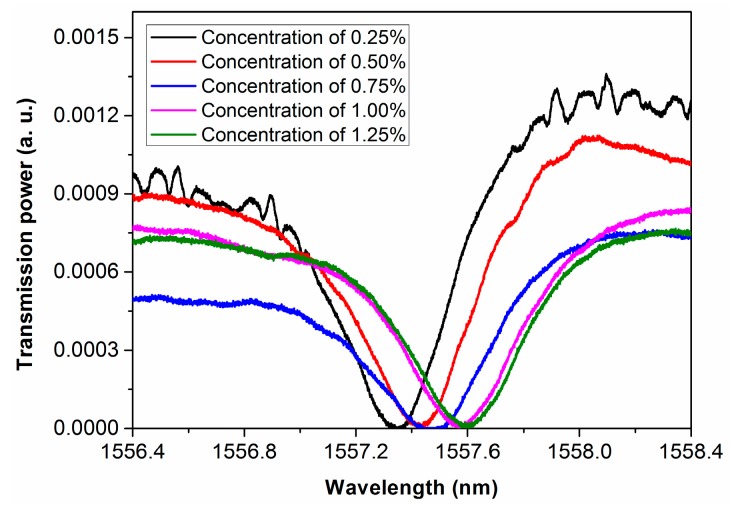
The close-up view of the spectral dips around 1557 nm wavelength measured at different doping concentrations of salt.

**Figure 14:**
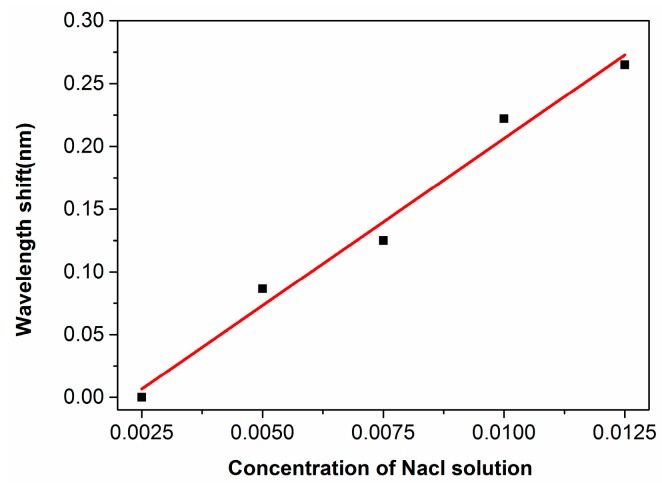
The wavelength shift plotted as a function of the doping concentration of salt (**black squares**), which can be well fitted with a linear relationship (**red curve**).

Figure 13 shows five spectra recorded in purified water doped with salt at several different concentrations of 0.25%, 0.5%, 0.75%, 1%, and 1.25%. One can see a dramatic shift of the spectral dip around 1557 nm wavelength with the increasing concentration of the salt in water, which is a result of the varying refractive index of water at various doping concentrations of salt. In addition, Figure 14 shows that the position of the spectral dip shifts almost linearly as a function of the doping concentration of salt, which is reasonable as at such low concentrations of the salt, the refractive index of the liquid will vary linearly with the doping concentration. Based on the data in Figure 14, we have evaluated that the sensitivity of our sensor can reach ~220 nm/RIU, yielding a detection limit of 1.2 × 10^−4^ RIU. The results are comparable to the best sensors fabricated by the FLDW so far. It should be mentioned that in such high-sensitivity measurements, the temperature in the sensor must be highly controlled to avoid the refractive index fluctuation induced by temperature variation [28].

### 3.3. Integrated Microfluidic Chip for Electrophoresis Analysis

In 2013, Ju et al. demonstrated a fully functional capillary electrophoresis (CE) analysis chip directly fabricated in glass using FLDW [29]. Electrophoresis has been recognized as an important approach in chemical and biological analysis. Specifically, CE achieves separation of charged analytes with different size-to-charge ratios by accelerating them in thin capillaries under the influence of an electric field. The analytes will be accelerated to different velocities so as to be spatially redistributed in different regions in the capillaries. It has been realized that the use of microfluidic CE chips will lead to compact and portable device which consumes only picoliters of samples and produce less waste [1].

**Figure 15:**
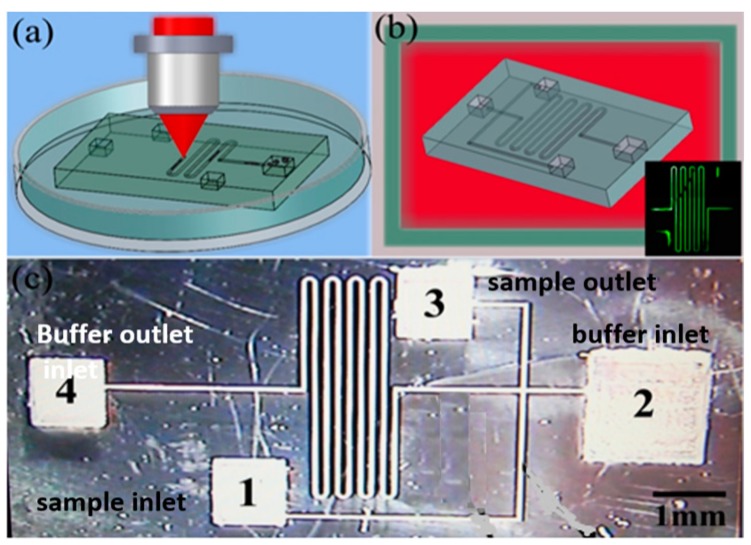
Flow chart of fabrication procedures: (**a**) laser direct writing inside porous glass and (**b**) annealed in a furnace. Inset: fluorescence microscopy image of the microchannel filled with a solution of fluorescein; (**c**) optical micrograph of the fabricated electrophoresis microchip.

The CE chip was fabricated in the porous glass using the technique discussed in Section 2.1, as briefly shown in the flow chart illustrated in Figure 15a,b. The completed CE microchip is presented in Figure 15c. The smooth edge of the channel indicates that the ablation debris generated during the laser writing has been efficiently removed. The lengths of the longer (migration) and shorter (sampling) microchannels are 35 mm and 8 mm, respectively. In addition, the channels have a depth of ~10 μm and a width of ~36 μm. The depth and width of the channels were controlled by optimizing the focusing condition as well as the scan configuration.

**Figure 16:**
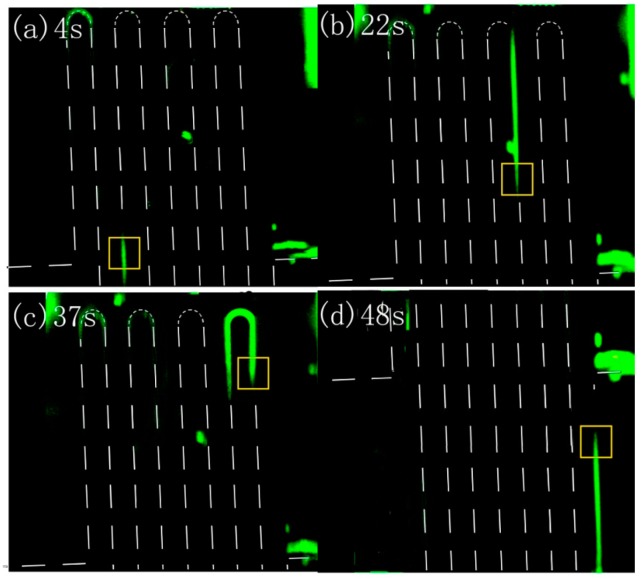
Fluorescence microscopy images of the derivatized amino acids positions at different times with a voltage of 620 V applied across the migration channel and 200 V across the sampling channel. (**a**) 4 s; (**b**) 22 s; (**c**) 37 s; (**d**) 48 s.

The CE separation function of the femtosecond-laser-fabricated microchip was demonstrated by observing the migration of derivatized amino acids in the microfluidic channels with a constant velocity, as shown in Figure 16. After the derivatized amino acids were injected into the inlet, a voltage of 200 V was applied across the sampling channel, whereas the voltage applied across the migration channel was 100 V. In 2 s, the amino acids were driven into junction of the two microchannels. With a migration voltage of 620 V further applied across the migration channel and a voltage of 200 V across the sampling channel, migration of amino acids was observed under a fluorescence microscope (IX71, Olympus, Tokyo, Japan). Images of the derivatized amino acids were captured at different times when the constant migration voltage was applied across the migration channel. The moving fronts of the amino acids at different times are indicated by the yellow box in Figure 16. Clearly, amino acids migrated in the microfluidic channel, and the velocity of migration was evaluated to be 0.49 mm/s. The observed linear increase of the migration distance with the increasing time confirms the homogeneity of the fabricated migration channel.

## 4. Conclusions and Future Perspective

FLDW has provided an attractive solution for fabricating micro- and nanofluidic structures of 3D geometries. To fulfill the requirements of real world applications with the 3D micro- and nanofluidic structures, challenges need to be overcome in terms of achieving large footprint sizes, nanoscale fabrication resolutions, and low surface roughness. Several technical solutions have been discussed in this review. Furthermore, we show several examples of integrated chemical and biological chips fabricated by FLDW, and demonstrate their functionalities including SERS-based molecular sensing, whispering gallery mode (WGM)-based refractive index sensing, and CE analysis of derivatized amino acids. 

The future of the technique relies on exploration of innovative applications by fully utilizing the unique capabilities of manipulation of fluidic dynamics in 3D space as well as that of reduction of channel dimensions to the level of single molecules, both of which are offered by FLDW. In addition, the nearly unlimited flexibility of incorporating various types of functionalities ranging from microoptics and photonics to microelectrodes and plasmonics into the microfluidic systems will lead to smaller and smarter sensing microdevices. One challenge that has not been seriously considered is the realization of multi-scale structures in one-step continuous direct writing, in which the feature dimensions can span three or four orders of magnitude. For example, a microfluidic system may consist of a chamber on the millimeter or even centimeter scale and an array of nanochannels with a uniform transverse size on the submicrometer scale. Although FLDW can provide the resolution for fabricating the submicrometer-scale channels with a high numerical aperture objective, it will be extremely time-consuming to produce the large chamber at the same fabrication resolution. One solution is to shape the focal spot size spatially and temporally, which can allow for dynamically switching the focal volume size (i.e., equivalent to switching the fabrication resolution) during the fabrication process whilst still ensuring a 3D isotropic resolution even when the numerical aperture of the focusing system varies [30]. The field will remain active as long as better performances and higher functionalities of 3D micro- and nanofluidics are highly in demand for emerging innovative applications.
